# Treatment‐Free Remission in Chronic Myeloid Leukemia: Revisiting the “W” Questions

**DOI:** 10.1111/ejh.70000

**Published:** 2025-06-23

**Authors:** Antonella Bruzzese, Ernesto Vigna, Enrica Antonia Martino, Nicola Amodio, Caterina Labanca, Francesco Mendicino, Eugenio Lucia, Virginia Olivito, Fortunato Morabito, Massimo Gentile

**Affiliations:** ^1^ Hematology Unit, Department of Onco‐Hematology AO of Cosenza Cosenza Italy; ^2^ Department of Experimental and Clinical Medicine University of Catanzaro Catanzaro Italy; ^3^ AIL Sezione di Cosenza Cosenza Italy; ^4^ Department of Pharmacy, Health and Nutritional Science University of Calabria Rende Italy

**Keywords:** CML, TFR, TKI

## Abstract

Chronic myeloid leukemia (CML) has undergone a transformation from a fatal disease to a chronic, manageable condition with the advent of tyrosine kinase inhibitors (TKIs), particularly imatinib. This shift has significantly improved survival rates, and for some patients, achieving deep molecular response (DMR) has made treatment‐free remission (TFR) a feasible goal. However, the ability to sustain TFR remains a challenge, primarily due to the persistence of leukemia stem cells (LSCs), which are inherently resistant to TKIs and contribute to relapse after treatment cessation. The barriers to TFR include not only the persistence of LSCs but also the complex biology of resistance mechanisms. Attempts to overcome these barriers through TKI rotation, second‐generation TKIs, or strategies targeting the tumor microenvironment have shown limited success. Third‐generation TKIs like ponatinib and novel agents such as asciminib and olverembatinib offer promising therapeutic alternatives, particularly for patients with the T315I mutation. However, their use is constrained by safety concerns and limited availability, especially in low‐resource settings. Patient selection remains a crucial aspect of achieving TFR, as not all patients with DMR sustain remission after discontinuation of therapy. Factors such as age, immune profile, and measurable residual disease levels significantly influence relapse risk. Identifying reliable clinical and molecular predictors of successful TFR is essential for optimizing treatment strategies and improving outcomes. The concept of the “W” questions—who, when, why, what, and where—frames the future of TFR in CML. Who should be selected for TFR? When should TFR be pursued? Why are some patients successful in achieving and sustaining TFR, while others relapse? What strategies can enhance the likelihood of TFR success, such as novel therapies, combination treatments, and patient‐specific approaches? Where should these strategies be implemented to ensure equitable access, particularly in resource‐limited settings? This review highlights the progress made in CML treatment, emphasizes the need for precision medicine to personalize TFR strategies, and stresses the importance of answering the fundamental “W” questions to advance the field. Future research must focus on refining predictive models, exploring new therapeutic options, and expanding access to treatments to ensure equitable benefits for all CML patients globally.

## 1 Introduction

Chronic myeloid leukemia (CML) is a clonal hematopoietic stem cell malignancy characterized by the uncontrolled proliferation of myeloid cells, predominantly affecting the granulocytic lineage [1, 2, 3]. Without treatment, CML typically progresses from a chronic phase (CP) to an accelerated phase (AP) and ultimately to blast crisis (BC), a stage resembling acute leukemia [1, 2, 3]. CML is rare, with an estimated annual incidence of approximately 1–2 cases per 100 000 individuals in Western countries, a median age at diagnosis of 65 years, and a slight male predominance. Clinical presentation is variable: while many patients frequently report non‐specific symptoms, nearly 40% of the patients are diagnosed incidentally through routine laboratory evaluations revealing unexplained leukocytosis. Splenomegaly is the most common physical finding, present in approximately half of newly diagnosed patients [1, 2, 3, 4].

CML is driven by the t(9;22) (q34;q11) translocation, resulting in the formation of the Philadelphia (Ph) chromosome and the *BCR::ABL1* fusion gene. This produces a constitutively active tyrosine kinase that drives proliferation, blocks apoptosis, and induces genomic instability, key features of CML leukemogenesis [4]. In ~5% of the cases, the Ph chromosome is cryptic or undetectable by standard cytogenetics; diagnosis therefore relies on FISH or RT‐PCR, which detects the BCR::ABL1 transcript. These patients have a similar prognosis and response to therapy as those with cytogenetically visible Ph + CML [3, 4].

CML progression is largely attributed to the genetic instability imparted by BCR::ABL1, which facilitates the acquisition of additional cytogenetic and molecular abnormalities that drive transformation from CP to AP and BC [4, 5, 6].

The advent of tyrosine kinase inhibitors (TKIs) revolutionized CML management. Imatinib, the first BCR::ABL1‐targeted agent, demonstrated remarkable efficacy in the IRIS trial, leading to the development of second‐ and third‐generation TKIs (dasatinib, nilotinib, bosutinib, and ponatinib) with greater potency and mutation coverage. These advances have significantly improved outcomes, with CP‐CML patients now having a life expectancy comparable to the general population in most Western countries [7, 8, 9, 10, 11, 12, 13, 14, 15, 16, 17, 18].

Shanmuganathan et al. critically reviewed the comparative efficacy, safety, and treatment goals associated with first‐line TKIs in newly diagnosed CML‐CP patients [19]. Three main strategies should guide TKI selection, each balancing efficacy, safety, and cost. The choice depends on patient characteristics, healthcare resources, and therapeutic goals such as deep molecular response (DMR) and treatment‐free remission (TFR). Starting with a second‐generation TKI offers the highest chance of rapid and deep response, translating into greater TFR potential. Using imatinib first is cost‐effective and widely adopted, especially in settings with limited resources. However, it may delay or reduce the chance of achieving TFR. A third option, beginning with a potent TKI and then switching to a less toxic or cheaper agent after response, aims to combine efficacy with long‐term tolerability and affordability. While promising, this approach remains experimental [7, 8].

Overall, while second‐generation TKIs (2G‐TKIs) used upfront offer the best path to TFR [7, 8, 15, 16, 17], the optimal approach should be tailored to the patient's clinical context and treatment goals.

As TFR becomes more feasible, research has focused on factors influencing its outcome—including leukemic stem cell (LSC) persistence, BCR::ABL1‐independent survival, immune function, and the bone marrow microenvironment [19].

This review examines the biological basis, clinical evidence, and future directions of TFR in CML, structured around the “W questions” (Who, What, When, Where, Why, and How) to offer a comprehensive overview of its potential and challenges.

## 2 Who: Identifying Candidates for TFR

The achievement of major molecular response (MMR, BCR::ABL1 ≤ 0.1% on the International Scale [IS]) is a pivotal early goal in the management of CML. MMR correlates with improved long‐term outcomes, including progression‐free and overall survival (PFS and OS), and is a prerequisite for subsequent attempts at TFR. In patients treated with TKIs, failure to reach MMR within 12 months is considered a warning sign and is associated with an increased risk of disease progression, prompting consideration of therapeutic modification.

Molecular response categories according to ELN recommendations are summarized in Table 1.

**TABLE 1:** Scoring molecular response according to ELN recommendation.

	MR3 (MMR)	MR4	MR4.5	MR5
Minimum sum of reference gene transcripts	10 000 ABL1 24 000 GUSB	10 000 ABL1 24 000 GUSB	32 000 ABL1 77 000 GUSB	100 000 ABL1 240 000 GUSB
BCR‐ABL1 transcript level on the IS	≤ 0.1%	≤ 0.01	≤ 0.0032	≤ 0.001

Abbreviation: IS, international scale.

TFR, the ability to maintain remission after stopping TKIs, is emerging as a feasible goal in selected patients. While deeper molecular responses such as MR4 or MR4.5 are often prerequisites for attempting TFR, MMR remains a key surrogate marker that supports molecular stability and serves as a baseline for monitoring molecular recurrence [7, 8, 20, 21, 22, 23, 24, 25, 26]. The STIM1 trial, one of the first to investigate TFR feasibility, enrolled 100 chronic‐phase CML patients with sustained undetectable BCR::ABL1 transcripts (undetectable measurable residual disease, MRD) for at least 2 years on imatinib. At a median follow‐up of 77 months, the estimated treatment‐free survival (TFS) was 39% at 5 years. Importantly, molecular relapse (defined as loss of MMR) occurred in 61% of the patients, with most relapses (80%) within the first 6 months of discontinuation. All relapsed patients regained MMR after restarting imatinib, highlighting the safety of discontinuation in patients with sustained DMRs [27, 28].

Building on these findings, the EURO‐SKI study—the largest TFR trial to date—analyzed 755 CML patients treated with any TKI (most commonly imatinib) for at least 3 years, with sustained MR4 for at least 1 year. The TFR at 6, 24, and 36 months were 61%, 50%, and 46%, respectively. Loss of MMR was used as the criterion for molecular relapse and occurred primarily within the first 6 months following TKI discontinuation. Patients with longer durations of both TKI treatment duration and MR4 had better outcomes. A predictive model developed from EURO‐SKI data identified the duration of TKI treatment, duration of MR4, and BCR::ABL1 transcript type (e13a2 vs. e14a2) as significant predictors of successful TFR. Additionally, the risk of late relapse was associated with the percentage of blasts in peripheral blood, TKI treatment duration, and platelet count at diagnosis [29, 30].

Second‐generation TKIs, such as nilotinib and dasatinib, induce deeper and faster molecular responses than imatinib, prompting studies evaluating their role in facilitating TFR [15, 16, 17]. The ENESTfreedom trial investigated TFR in newly diagnosed chronic‐phase CML patients who achieved MR4.5 on first‐line nilotinib. Among the 190 patients who entered the TFR phase after 2 years of nilotinib consolidation, the 1‐year TFS was 51.6%, with 93% of the relapsed patients regaining MMR within 13 weeks of restarting nilotinib. Similarly, the ENESTop trial evaluated TFR after switching from imatinib to nilotinib in patients who achieved sustained MR4.5. The 48‐week TFS was 57.9%, and nearly all patients regained MMR upon retreatment [31, 32, 33]. The DASFREE study confirmed comparable findings with dasatinib: of 84 patients who discontinued after achieving sustained DMR, 49% remained in TFR at 2 years, with molecular relapse primarily defined as loss of MMR and occurring early [34]. Collectively, these studies reinforce that loss of MMR is a sensitive and safe trigger for TKI resumption and that MMR serves as a reliable threshold for molecular relapse across TKI discontinuation trials.

Beyond clinical trials, real‐world data from registries and observational cohorts support the safety and reproducibility of TFR. In the RE‐STIM study, a retrospective analysis of patients who attempted a second TKI discontinuation and loss of MMR again served as the relapse definition, and the re‐achievement of MMR upon TKI resumption was consistently high [35]. Meta‐analyses have further solidified MMR's role. A systematic review of 10 TFR studies, including STIM, EURO‐SKI, ENESTfreedom, ENESTop, and DASFREE, found that the median molecular recurrence‐free survival was approximately 50% at 24 months across studies using loss of MMR as the relapse criterion. These data demonstrate the uniformity of defining relapse as MMR loss and confirm the high rate of response recovery after TKI reinitiation [36]. A retrospective Italian study reported a 12‐month TFR rate of 68%, with higher relapse rates observed in younger patients and in those who discontinued treatment after more advanced lines of therapy [37].

In virtually all major trials (including STIM1, EURO‐SKI, STOP 2G‐TKI, ENESTfreedom, ENESTop, DADI, and DASFREE), relapse after TKI discontinuation occurs predominantly within the first 6 months and is driven by loss of MMR. The DADI trial, conducted in Japan, reported that 44% of the patients lost MMR within the first 6 months after stopping dasatinib, but 98% regained MMR within 3 months of resuming therapy. Likewise, the D‐FREE study, which evaluated early discontinuation of dasatinib after only 1 year of consolidation, showed that insufficient duration of DMR increased relapse risk, with MMR loss as the primary trigger for restarting treatment. The JALSG‐STIM213 study reported similar findings, confirming that sustained DMR duration and longer TKI exposure were associated with higher TFR success [27, 38, 39, 40, 41, 42, 43].

Additional confirmation comes from ENESTfreedom and ENESTop, in which molecular recurrence occurred in 49%–57% of the patients but was reversible in over 90% of the cases following nilotinib reintroduction. Long‐term follow‐up of ENESTfreedom showed that of 190 patients who entered the TFR phase, 44% maintained TFR at 5 years, and only 2 patients experienced disease progression, both with identifiable risk factors. Most relapses occurred early and were marked by loss of MMR, and all patients who restarted nilotinib regained MMR rapidly [31, 32, 33].

These findings consolidate MMR's utility as a clinically meaningful threshold for monitoring during and after TKI discontinuation. MMR offers a reliable and standardized measure to define molecular recurrence, guide safe treatment reintroduction, and counsel patients on relapse risk. Moreover, the high rates of MMR recovery across all studies emphasize the biological reversibility of molecular relapse, provided that monitoring is frequent, especially within the first 6 months of TFR. The consistent use of MMR as a benchmark across diverse populations and TKI types underscores its centrality to TFR protocols. In clinical practice, achieving and sustaining MMR remains an essential prerequisite for considering TFR. While deeper responses such as MR4 or MR4.5 are required for entry into TFR, the maintenance or loss of MMR is the decisive factor in long‐term treatment decisions. When a lower threshold of response was used for TFR eligibility, as in the DESTINY study, patients without DMR had a greater proportion of MMR loss [43].

Achieving TFR is a key aspiration for many patients, provided it can be done safely. However, with imatinib, long‐term TFR remains elusive for the majority, with success rates generally under 30% [27, 28, 29, 30, 31, 32, 33, 34, 35, 36, 37, 44, 45].

Although robust long‐term TFR data for 2G‐TKIs are still limited, their ability to induce faster and more sustained DMRs, along with encouraging early TFR outcomes, suggests that durable remission off therapy may become more common even when 2G‐TKIs are used as second‐line treatment, as demonstrated in the ENESTop, DADI, and DASFREE studies [31, 32, 33, 34, 35, 40, 41, 45]. An additional advantage of 2G‐TKIs is that patients can become eligible for TFR after a shorter overall treatment duration. Ongoing frontline trials of asciminib, all of which include TFR arms, are expected to shed more light on the potential of this agent to increase the proportion of patients who can attempt TFR [NCT06236724, NCT06368414NCT04838041].

## 3 What: Understanding the Mechanisms of Failure

TFR in CML reflects deep molecular control and the potential for drug discontinuation. However, its durability is often compromised by the persistence of neoplastic clones despite apparent disease suppression. These mechanisms, both BCR::ABL1‐dependent and ‐independent, play a crucial role in relapse and therapeutic failure.

### 3.1 Leukemic Stem Cells

The persistence of LSCs plays a crucial role in TFR failure. However, a direct correlation between LSC burden and BCR::ABL1 transcript has not been demonstrated. Bocchia and colleagues reported that CD26+ LSCs persist in the majority of CML patients who are in molecular response while receiving TKI treatment, and even after TKI discontinuation, without any correlation between BCR::ABL1 levels and the number of residual LSCs [46].

Moreover, leukemic cells may activate alternative signaling pathways—such as JAK/STAT, PI3K/AKT, RAS/MAPK, and SRC—which sustain survival under TKI pressure. These pathways also induce epigenetic modifications, including histone methylation, acetylation, and DNA methylation, to maintain LSC persistence [47, 48, 49, 50].

The Hedgehog (Hh) signaling pathway is aberrantly activated in CML and is essential for maintaining the self‐renewal capacity of LSC. Inhibition of Smoothened (Smo), a key component of the Hh pathway, has been shown to reduce the expansion of imatinib‐resistant LSCs. Furthermore, dual inhibition of Hh and BCL‐2 pathways sensitizes CML cells to imatinib, enhancing therapeutic efficacy [51, 52, 53].

### 3.2 The Role of the Immune System

The immune system's role in achieving and maintaining DMR, particularly after TKI discontinuation, remains incompletely understood. Before TKIs, therapies like interferon‐α enhanced natural killer (NK) cell cytotoxicity in CML patients [54]. Recent studies show that NK cell function may be relevant even during TKI therapy. Patients who achieve MMR or MR4.5 often exhibit a more differentiated NK‐cell phenotype, suggesting ongoing immune surveillance. Higher NK cell counts correlate with sustained remission following TKI cessation [55, 56].

The graft‐versus‐leukemia (GvL) effect in allo‐HSCT also emphasizes the importance of immune surveillance in CML, controlling disease recurrence even in measurable residual disease [7, 8, 57, 58, 59]. However, immunotherapeutic strategies aiming to prolong TFR have shown mixed results. For example, the ENDURE, CML‐IX trial found no significant improvement in TFR success with ropeginterferon treatment, indicating that boosting Th1 immune responses alone may not be sufficient for long‐term disease control [60].

Moreover, the immunogenicity of CML cells suggests a possible role of vaccination to improve response and TFR success. Yong et al. suggested that peptide‐based vaccines targeting WT1 in combination with PRAME, could additionally improve targeting differentiated progeny, improving response rates [61, 62]. Recently 2 multicenter phase 2 studies (GIMEMA CML0206 and SI0207) showed the feasibility, safety, no off‐target events p210 peptide vaccinations in CML patients with persisting disease during imatinib treatment, reporting also a 16.5% rate of TFR by 48 months [63].

### 3.3 The Bone Marrow Microenvironment Role

The bone marrow microenvironment (BMME) plays a critical role in LSC persistence and TFR maintenance in CML. The CXCL12–CXCR4 axis facilitates LSC homing and retention in the protective bone marrow niche, contributing to TKI resistance. Targeting CXCL12 with NOX‐A12 has shown synergistic effects with TKIs in vitro. Additionally, the enzyme CD26, expressed on CML LSCs, modulates leukemic cell survival by cleaving CXCL12 and enabling immune evasion. Inhibition of CD26 with gliptins reduces stromal interactions and offers a therapeutic strategy.

Moreover, CXCL1–CXCR2 signaling, upregulated in CML, sustains LSC self‐renewal and quiescence, presenting another therapeutic vulnerability [64, 65, 66, 67]. Adhesion molecules like CD44 and vascular E‐selectin contribute to LSC retention in the niche. Inhibition of these molecules enhances apoptosis and improves survival in preclinical models [68, 69, 70].

Exosome‐mediated crosstalk between CML cells and the BMME also modulates disease progression and resistance. CML‐derived exosomes can impair T‐cell function and foster an immunosuppressive environment [71, 72, 73, 74, 75]. These findings underscore the BMME's active role in CML pathobiology, suggesting that disrupting BMME–LSC interactions could improve TFR outcomes and eradicate residual disease.

## 4 When: Timing of TFR

One of the most debated and clinically relevant questions in the field of CML today concerns the optimal duration of DMR required to maximize the chances of successful TFR. Although major clinical trials and expert consensus statements often reference a minimum of 2 years of sustained DMR before attempting TKI discontinuation, it has become increasingly clear that this is not a rigid threshold but rather a pragmatic guideline [7, 8].

Indeed, the evidence consistently suggests that longer durations of DMR are associated with a higher probability of sustained TFR. Data from pivotal studies such as EURO‐SKI and ENESTfreedom demonstrate that patients who maintained MR4 or deeper for three or more years before discontinuation had a significantly reduced risk of molecular relapse [29, 30, 31, 32, 33].

Bonifacio et al. further reinforced this observation by showing that patients with “non‐optimal” DMR durations (less than 3 years of MR4 or less than 2 years of MR4.5) still achieved TFR in more than half of the cases, while those who met or exceeded these thresholds experienced TFR rates approaching 78% [76].

What emerges from these findings is that while a minimum DMR duration of 2 years provides a reasonable baseline for clinical decision‐making, it should not be viewed as an absolute requirement. Rather, the duration of DMR should be interpreted in the context of each patient's molecular response kinetics, adherence history, comorbidities, and psychological readiness. Importantly, close molecular monitoring remains essential, particularly in the first 6 months after TKI cessation, when the risk of relapse is highest.

While multiple studies have demonstrated that a longer duration of imatinib therapy is significantly associated with improved TFR outcomes, the same relationship appears less consistent when applied to 2G‐TKIs. For instance, data from the STIM1, TWISTER, and EURO‐SKI trials have shown that extended exposure to imatinib before cessation serves as a protective factor against molecular relapse following TKI withdrawal [27, 28, 29, 30, 44]. In the case of dasatinib, the DASFREE study similarly reported enhanced TFR durability among patients with longer treatment durations. Conversely, in trials such as ENESTfreedom and the first‐line DADI study, the duration of TKI treatment did not significantly correlate with TFR success [31, 32, 33, 34, 40, 41, 42, 43]. These findings collectively suggest that the impact of treatment duration on TFR may be agent‐specific, differing between imatinib and 2G‐TKIs.

Additional real‐world data have provided further insights into dose modifications and their influence on TFR outcomes. A retrospective Chinese study evaluated 190 patients who discontinued TKI therapy, including 27 individuals with a prior dose reduction. TFR rates at 6, 12, and 24 months were 76.9%, 68.8%, and 65.5%, respectively. Notably, there was no statistically significant difference in 24‐month TFR rates between patients who had undergone prior dose reductions and those who had not (55.8% vs. 66.7%), indicating that dose reductions do not negatively affect the likelihood of maintaining TFR [77].

Similarly, an Italian multicenter retrospective analysis involving 1785 CML patients treated with reduced‐dose TKIs (primarily due to comorbidities or adverse events) demonstrated that TFR could be achieved in 248 patients (13.9%), nearly all of whom were in confirmed DMR at the time of discontinuation. With a median follow‐up of 24.9 months, 172 patients (69.4%) remained in TFR. Predictors of TFR success included the absence of prior resistance to any TKI, longer DMR duration before discontinuation, and the presence of the e14a2 BCR::ABL1 transcript variant. Conversely, variables such as gender, prior interferon exposure, Sokal/ELTS risk scores, TKI type, reason for dose reduction, and total TKI treatment duration did not significantly influence TFR outcomes. These results underscore the notion that it is not the absolute duration of therapy but rather the duration and stability of molecular response that most strongly predict TFR success [78].

This concept was further validated by a retrospective cohort study from the MD Anderson Cancer Center evaluating 284 patients who discontinued imatinib, dasatinib, or nilotinib. Multivariate analysis identified that only patients with a sustained MR4 or MR4.5 for at least 5 years before discontinuation experienced a significantly lower risk of molecular relapse. In contrast, a retrospective Spanish study did not find any clear association between TFR success and either treatment duration or response duration, highlighting some variability across cohorts and suggesting that additional predictive biomarkers are still needed [79, 80].

The GIMEMA CML 0307 trial analyzed 73 patients with CP CML treated initially with nilotinib 400 mg twice daily, later adjusted to 300 mg twice daily following regulatory approval. After a median treatment duration of 88 months, 24 patients (32.9%) who achieved stable DMR discontinued treatment. Among them, the estimated 2‐year TFS was 72.6%. When calculated across the entire study cohort, the overall TFR rate was 24.7% (18 of 73 patients) [81].

Collectively, these data suggest that while TKI treatment duration contributes to TFR success, especially with imatinib, it is the quality and persistence of the molecular response, rather than therapy length alone, that most reliably predicts favorable outcomes. Accordingly, current European LeukemiaNet (ELN) recommendations emphasize both depth and duration of molecular remission as prerequisites for TFR (criteria summarized in Table 2) [7]. A synthesis of the major clinical trials exploring TFR is provided in Table 3. Features associated with the major probability of TFR success are summarized in Table 4.

**TABLE 2:** Requirement for TKI discontinuation according to ELN recommendation.

Mandatory
CML in first CP only (data are lacking outside this setting)
Motivated patient with structured communication
Access to high‐quality quantitative PCR using the International Scale (IS) with rapid turn‐around of PCR test results
Patient's agreement to more frequent monitoring after stopping treatment. This means monthly for the first 6 months, every 2 months for months 6–12, and every 3 months thereafter.
Minimal (stop allowed)
First‐line therapy or second‐line if intolerance was the only reason for changing TKI
Typical e13a2 or e14a2 BCR–ABL1 transcripts
Duration of TKI therapy > 5 years (> 4 years for 2GTKI)
Duration of DMR (MR4 or better) > 2 years
No prior treatment failure
Optimal (stop recommended for consideration)
Duration of TKI therapy > 5 years
Duration of DMR > 3 years if MR4
Duration of DMR > 2 years if MR4.5

**TABLE 3:** Main studies exploring the TFR.

Name of study	Number of patients who discontinued TKI	TKI used	TFR success probability	References
STIM1	100	Imatinib	6 months 43% 5 years 38%	[27, 28]
TWISTER	40	Imatinib	2 years 47.1%	[44]
STP 2G‐TKI	60	Dasatinib or Nilotinib	1 year 65% 4 years 55.24%	[38, 39]
2nd line DADI	88	Dasatinib as 2nd line	3 years 44.4%	[40, 41]
1st line DADI	68	Dasatinib 1st line	6 months 55.2%	[42]
DASFREE	84	Dasatinib 1st line	2 years 46%	[34]
ENESTop	126	Nilotinib 2nd line	5 years 42.9%	[33]
ENESTfreedom	190	Nilotinib 1st line	4 years 51.6%	[31, 32]
EURO‐SKI	758	Imatinib or Dasatinib or Nilotinib	6 months 61% 2 years 50%	[29, 30]
DESTINY	MMR group 49 MR4 group 125	Imatinib or Dasatinib or Nilotinib	3 year MMR group 36% MR4 group 72%	[43]
GIMEMA CML 0307 study	24	Nilotinib	2 years 72.6%	[81]
ENESTPath	Arm A 119 Arm B 104	Nilotinib	1 year Arm 1 31.9% Arm 2 37.5%	[82]
PETALs	Arm A 21 Arm B 19	Arm A Nilotinib alone Arm B Nilotinib + Peg‐IFNα	na	[83]
TIGER	Experimental 153 Comparator 192	Experimental Nilotinib + Peg‐IFNα Comparator Nilotinib	1 year Experimental 69% Comparator 60% 2 years Experimental 57% Comparator 48%	[84]
Retrospective study	190	Imatinib or Dasatinib or Nilotinib or Flumatinib	6 months 76.9% 1 year 68.8% 2 years 65.5%	[85]
Retrospective study	236	Imatinib or 2 g‐TKIs	1 year 73.8%	[86]
Retrospective study	199	Imatinib or Dasatinib or Nilotinib or Bosutinib or Ponatinib	5‐years 79%	[83]

Abbreviations: TFR, treatment‐free remission; TKI, tyrosine kinase inhibitor.

**TABLE 4:** Features associated with better TFR outcomes and relative studies.

Factors predictive of TFR success	Relative reference
Longer duration of treatment	[27, 28, 29, 30, 40, 41, 44]
Longer duration of DMR	[29, 30, 31, 32, 76, 78, 79]
Typical e13a2 transcript	[29, 30]
Typical e14a2 transcript	[78]
Older age	[37]
First line of treatment	[37]
Achievement of DMR instead of MMR	[43]
Lower CD4+ cells at discontinuation	[42]
Absence of prior TKI resistance	[78]

## 5 Where: Drug Accessibility in Low‐Income Countries

TFR in CML is strongly influenced by geographic and socioeconomic disparities, especially in low‐ and middle‐income countries (LMICs), where access to diagnostics, therapies, and monitoring is limited. Patients in these regions often present with high‐risk diseases and face systemic barriers such as drug scarcity and inadequate molecular monitoring. A global registry of 1853 newly diagnosed CML patients highlighted substantial gaps in guideline‐based care, particularly in Latin America, Asia‐Pacific, the Middle East, and Africa. Hydroxyurea was the initial treatment in 61.9% of the cases, while only 29.5% began imatinib; this increased to 81.1% during follow‐up, but second‐generation TKIs were rarely used [87].

LMICs often lack the infrastructure for BCR::ABL1 transcript monitoring, essential for guiding TFR strategies. Rowley et al. estimated a $29.1 million gap in PCR monitoring capacity across 60 LMICs over 5 years, underscoring the magnitude of this unmet need [88].

Nonetheless, effective care is possible even in low‐resource settings. In rural Rwanda, decentralized district hospitals supported by public‐private partnerships and national insurance achieved favorable outcomes [89], demonstrating that guideline‐concordant care is feasible when infrastructure is in place.

A study by Casey et al. revealed a stark mismatch between disease burden and research activity: while South Asia and Sub‐Saharan Africa bear a large share of CML‐related DALYs, they are almost absent from clinical trials. None of the 30 pivotal TKI trials included low‐SDI countries, and only one (India) represented the low‐middle SDI group [90].

This exclusion has broad implications. Low‐SDI countries, often with high fertility rates and growing CML incidence in younger populations, face the greatest barriers to clinical trial participation, limited research infrastructure, regulatory hurdles, and underinvestment. Consequently, drug development largely overlooks the populations most in need.

Moreover, while high‐SDI countries have access to multiple TKIs, low‐SDI countries often rely on just one, restricting options in cases of resistance or intolerance. Third‐generation agents like ponatinib and asciminib, vital for high‐risk patients, are largely unavailable in LMICs.

Generic TKIs, though improving affordability, introduce concerns about efficacy and safety due to variable manufacturing quality and weak pharmacovigilance. The success of TFR hinges on uninterrupted access to high‐quality drugs and reliable molecular monitoring, both frequently lacking in LMICs.

TFR, increasingly a standard of care in high‐income countries, remains out of reach in LMICs. It requires not just sustained drug access but also molecular diagnostics, trained personnel, and systems for relapse detection and retreatment. Without these, discontinuation may risk relapse or progression.

Exclusion from clinical trials also undermines scientific validity. Pharmacogenomic variability, environmental factors, and comorbidities may influence TKI response and toxicity, making diverse representation essential not just ethically but scientifically.

Casey et al. call for structural changes in research priorities: broader TKI access, validation of generics, investment in diagnostics, and context‐adapted guidelines [90]. Without such reforms, TFR will remain a goal attainable only by a privileged few. Achieving global equity in CML care is not just a scientific challenge, it is a public health imperative.

## 6 Why: The Rationale for TFR

The rationale for TFR in CML is multifaceted, encompassing both clinical and patient‐reported outcomes. Although TKIs, especially imatinib, are generally well tolerated, second‐ and third‐generation agents, despite their superior potency, are linked to significant AEs that can affect morbidity and health‐related quality of life (HRQoL). Nilotinib increases the risk of arterial occlusive events and metabolic issues; dasatinib is associated with pleural effusion and pulmonary hypertension; and ponatinib carries a high risk of vascular toxicity. Up to 30% of the patients on long‐term TKIs experience AEs severe enough to require hospitalization or impair daily life, thus reducing HRQoL. The toxicity of TKIs has never been the primary endpoint of clinical studies, and a comparative evaluation of the frequency and the severity of toxicities between different TKIs is difficult. In all clinical trials, hematologic AEs are usually mild to moderate and limited to the first treatment period. Sometimes, they need a dose reduction but very rarely cause treatment discontinuation. Conversely, non‐hematologic AEs comprise a wide range of complications that affect quality of life or sometimes determine important morbidity (rarely mortality) as ischemic events with ponatinib, pancreatitis with nilotinib, or pleural effusion with dasatinib [7, 14, 15, 16, 17].

An Italian study found that younger CML patients (18–39 years) reported greater impairment in physical and emotional functioning, particularly in domains affected by physical and psychological symptoms. In contrast, older patients (> 60 years) had HRQoL scores closer to population norms. Women consistently reported lower HRQoL than men [91], highlighting the need for individualized treatment goals.

Patient attitudes toward TFR mirror these concerns. In a survey of 329 CML patients, 34% expressed a willingness to attempt TFR. The main motivations included side effects (56%), cost (52%), lifestyle impact (42%), and pregnancy desire (41%). Twenty‐six percent of the patients included in this study experienced a dose reduction for side effects, and 38% forgot to take any tablets in the last month, underlying the difficulty (especially for younger and active people) of being strictly adherent to a continuous treatment. Younger age, shorter disease duration, and higher symptom burden independently predicted favorable views on TFR [92].

TFR may also alleviate the economic burden of lifelong TKI therapy, especially as disease prevalence increases due to improved survival. In fact, treatment discontinuation not only reduces the direct costs but also decreases the economic burden associated with managing TKI‐related complications. Lowering long‐term drug exposure may reduce the incidence of cardiovascular and metabolic adverse events, which is particularly relevant for patients expected to remain on therapy for decades. Furthermore, discontinuation also leads to reduced laboratory and imaging monitoring requirements for treatment‐related toxicities, contributing to overall cost savings.

While TFR requires rigorous molecular monitoring, its value extends beyond molecular targets. It reflects a patient‐centered approach focused on improving quality of life, autonomy, and long‐term well‐being. Thus, TFR represents both a scientific advance and a shift toward aligning therapy with patient priorities and broader healthcare goals.

## 7 How: Strategies to Improve TFR

Achieving TFR requires a multifaceted approach that includes optimizing initial therapy, ensuring patient adherence, and utilizing combination strategies to overcome resistance. The integration of novel agents and combination therapies targeting alternative pathways are promising strategies to enhance treatment efficacy and expand the pool of patients eligible for TFR. Personalized medicine approaches, leveraging genetic and molecular profiling, are critical for tailoring treatment regimens to individual patient profiles and combination therapy approaches to address TKI resistance in the setting of residual leukemia.

### 7.1 Novel BCR::ABL1 Inhibitors and Therapeutic Combinations

Asciminib, a first‐in‐class STAMP inhibitor targeting the ABL myristoyl pocket, has demonstrated a novel, allosteric mechanism of action distinct from ATP‐competitive TKIs, offering new opportunities for achieving deep and sustained molecular responses in CML. In the phase 3 ASC4FIRST trial, asciminib showed superior efficacy to investigator‐selected TKIs in newly diagnosed patients, with an MMR at 48 weeks reaching 67.7% versus 49.0% overall and 69.3% versus 40.2% compared with imatinib alone. While the difference in MMR between asciminib and second‐generation TKIs (66.0% vs. 57.8%) was not statistically significant, asciminib led to faster and deeper responses: median time to MMR was 24.3 versus 36.4 weeks, and transcript levels ≤ 1% were achieved at 12.3 versus 24.0 weeks, respectively. Notably, 34.0% of the patients achieved a BCR::ABL1 transcript ≤ 0.01% and 18.4% achieved MR4.5, highlighting asciminib's potential in enabling TFR [93].

Crucially, the unique allosteric mechanism of asciminib opens opportunities for combination strategies. Its non‐overlapping binding site enables synergistic inhibition when paired with ATP‐competitive TKIs. A phase 1 study evaluated asciminib combinations with imatinib (ASC + IMA), nilotinib (ASC + NIL), or dasatinib (ASC + DAS) in CML‐CP/AP patients with ≥ 2 prior TKIs or T315I mutation. MMR at 96 weeks was 45.0% with ASC + IMA and 46.2% with ASC + DAS, despite the heavily pretreated cohort. ASC + IMA emerged as the most balanced option, with favorable efficacy and fewer serious adverse events. ASC + NIL had lower efficacy and a higher rate of arterial occlusive events (11.5%), while ASC + DAS showed more pleural effusion (12.5%) [94]. ASC + IMA achieved the maximum tolerated dose (60 mg asciminib + 400 mg imatinib) and is under further evaluation in ASC4MORE, which demonstrated higher rates of BCR::ABL1IS ≤ 0.0032% with no arterial events reported. Although combination therapy introduces the toxicity profiles of partner TKIs, most adverse events were manageable. ASC + IMA showed increased nausea and pancreatitis (12%), ASC + DAS had higher pleural effusion, and hematologic toxicities occurred across all regimens but were largely reversible [95].

While asciminib monotherapy remains a strong option in the third‐line setting and beyond, combination therapy, particularly with imatinib, may play a strategic role in selected patients aiming for deeper molecular response and ultimately TFR. Further prospective trials comparing monotherapy with combination strategies will be essential to better define their respective roles and to refine the selection of patient subgroups most likely to benefit from dual inhibition approaches.

Olverembatinib represents a major advancement in the therapeutic arsenal for CML, particularly for patients with resistance or intolerance to multiple TKIs [85]. It has demonstrated potent activity against a broad spectrum of BCR::ABL1 mutations, including the gatekeeper T315I mutation and compound variants, among the most formidable challenges in CML management.

In a phase 1/2 trial, olverembatinib induced robust and durable responses in patients with CP and AP CML previously exposed to ≥ 2 TKIs. Moreover, its safety profile appears more favorable than that of its predecessor, ponatinib, particularly regarding arterial occlusive events, which were infrequent despite cardiovascular adverse events occurring in approximately 32.1% of the patients. While these promising results require further validation, the high rates of molecular response suggest that olverembatinib may, in the future, contribute meaningfully to expanding the pool of patients eligible for TFR [86].

The pharmacokinetic profile of olverembatinib, compatible with alternate‐day dosing, adds convenience and may further support adherence and long‐term tolerability, key components in maintaining molecular remission and potentially enabling successful TFR.

Looking ahead, ongoing international studies (such as the POLARIS‐1 and ‐2 trials) are expected to provide critical data on olverembatinib's performance in newly diagnosed CML with or without T315I. These results will help determine its utility beyond salvage therapy and may even justify earlier‐line use in selected populations (NCT06423911).

Among other combination strategies, the PETALs study evaluated the impact of combining TKIs with immunomodulatory therapy on TFR outcomes in CP CML. Patients receiving first‐line therapy were randomized to either nilotinib monotherapy (Arm A) or to a sequential regimen consisting of Pegylated Interferon‐alpha (Peg‐IFN) for 30 days followed by a combination of nilotinib and Peg‐IFN for up to 2 years and then nilotinib alone (Arm B). At 12 months, the cumulative rate of MR4.5 was 15.9% in Arm A versus 21.5% in Arm B (*p* = 0.049), suggesting a modest advantage in molecular response depth with the combination strategy.

Moreover, patients in Arm B achieved sustained MR4.5 slightly earlier than those in Arm A (13 months vs. 16 months, respectively). In patients who experienced molecular relapse during TFR, nilotinib reintroduction successfully reestablished MMR within 6 months across both arms, with no observed differences in re‐sensitization rates [83].

Similarly, the TIGER study assessed the efficacy of combining nilotinib with Peg‐IFN. At 12 and 18 months, the probabilities of achieving MMR were 76% and 81% in the nilotinib monotherapy arm, compared to 83% and 88% in the combination arm, respectively. For MR4 at 18 months, the rates were 51% and 64% in the monotherapy and combination arms, respectively. However, analysis of the TFR cohort revealed that 24 months after treatment cessation, 22% of the patients in both the nilotinib and combination arms with typical BCR::ABL1 transcripts remained in TFR [84].

Collectively, these findings indicate that while the addition of Peg‐IFN to nilotinib enhances the speed and depth of molecular response, this does not translate into a significantly improved probability of long‐term TFR. The combination strategy may offer an early molecular advantage but does not appear to confer durable benefits in sustaining TFR, suggesting that factors beyond molecular response kinetics influence long‐term treatment discontinuation success.

### 7.2 Other Therapeutic Strategies

Therapeutic strategies targeting autophagy, whether genetically (e.g., ATG7 knockdown) or pharmacologically (e.g., hydroxychloroquine [HCQ], Lys05, PIK‐III), are gaining attraction as adjuncts to TKIs in the treatment of CML. Mitchell et al. demonstrated that co‐inhibition of mTOR and autophagy synergistically induced apoptosis in ponatinib‐resistant CML cells, highlighting the therapeutic vulnerability of autophagy‐dependent survival pathways [96]. Similarly, Baquero et al. demonstrated that autophagy inhibitors effectively target quiescent LSCs [97].

Although first‐generation autophagy inhibitors such as HCQ have demonstrated limited efficacy due to modest potency and off‐target effects, the biological rationale for targeting autophagy in CML remains robust, particularly for their potential to hit the LSCs that persist after treatment discontinuation despite the achievement of DMR [46, 47, 48, 49, 50, 98, 99].

Agents such as Lys05, a dimeric chloroquine derivative, and ULK1 inhibitors exhibit more potent and selective inhibition of autophagic flux, showing increased potential for synergistic interactions with TKIs and may help eradicate the therapy‐resistant stem cell reservoir. Moreover, dual targeting of BCR‐ABL signaling and autophagy has shown promising synergism in preclinical settings and is now advancing to clinical evaluation [100, 101, 102].

Another innovative approach is the repositioning of approved drugs with autophagy‐modulating properties. Compounds such as clarithromycin and celecoxib, which have well‐characterized safety profiles, modulate autophagy through diverse mechanisms, although their precise impact on LSC biology remains to be fully elucidated [103, 104]. Considering these findings, it is reasonable to propose that this combination be reserved for responder patients with persistent LSCs, with the aim of eradicating them and thereby enhancing the likelihood of successful TFR.

However, significant translational hurdles remain. Chief among them is the need for reliable pharmacodynamic biomarkers to monitor autophagy inhibition in real time. Given the dynamic and context‐dependent nature of autophagy, the lack of robust clinical assays for autophagy flux presents a major barrier to drug development and trial interpretation [105].

## 8 Discussion

The introduction of TKIs has transformed CML from a fatal disease into a chronic, manageable condition. Imatinib, in particular, revolutionized therapy by offering effective, well‐tolerated oral treatment. Over the past two decades, survival has improved markedly, and DMR has made TFR a feasible goal for some patients. However, challenges remain, especially in sustaining remission after discontinuation and overcoming resistance [7, 8].

A major barrier to TFR is the persistence of LSCs, which are intrinsically resistant to TKIs and contribute to relapse after treatment cessation. Targeting LSCs is therefore essential for improving TFR outcomes [46, 47, 48, 49]. While strategies such as DPPIV inhibition or combination therapies targeting STAT5/3 have shown promise, clinical results remain disappointing, reflecting the complexity of LSC biology [106, 107].

Attempts to overcome resistance via TKI rotation or early switch to second‐generation TKIs have had limited success, largely due to persistent LSCs [82, 108, 109, 110, 111]. This underscores the need to move beyond BCR‐ABL1 inhibition and address the tumor microenvironment, metabolic regulation, and epigenetics [46, 47, 48, 49, 50, 51, 52, 53, 54, 55, 56, 57, 58, 59, 60, 61, 62, 63, 64, 65, 66, 67, 68].

Patient selection for TFR remains critical. Although DMR is necessary, not all patients sustain remission after stopping therapy. Factors such as age, immune profile, and measurable residual disease levels influence relapse risk. Identifying clinical and molecular predictors is key to tailoring TFR strategies and minimizing relapse.

Third‐generation TKIs, especially ponatinib, provide options for patients with suboptimal responses or resistance, including those with the T315I mutation. Despite its potency, ponatinib's cardiovascular toxicity restricts frontline use [112, 113]. New agents like asciminib, targeting the ABL myristoyl pocket, showed promising efficacy and a better safety profile [93, 94, 95]. Olverembatinib is another emerging drug active against T315I and other mutations, with encouraging early data [85, 86]. While these therapies represent important advances, cost and limited availability may hinder access in low‐resource settings. Broader evaluation across populations will be essential to define their place in clinical practice.

Concerning the timing of BCR::ABL1 monitoring after discontinuation, the ELN guidelines suggest frequent monitoring and a structured follow‐up during this early period, with more intense surveillance during the first 6–12 months, typically involving monthly testing. Less intense monitoring after the first 6 months of discontinuation does not appear to increase the risk of progression or affect the success of TFR [114]. Further studies aimed at identifying patients more likely to maintain a safe TFR could help avoid over‐monitoring in those considered at lower risk of relapse, reserving monthly monitoring for patients at higher risk.

Moreover, the ELN recommends that molecular response be assessed according to the International Scale (IS) as the ratio of BCR‐ABL1 transcripts to ABL1 transcripts, or other internationally accepted control transcripts. Access to high‐quality quantitative PCR using the IS PCR test is mandatory before initiating TFR [7]. In recent years, novel technologies such as next‐generation sequencing (NGS) and digital PCR (dPCR) have been investigated; dPCR has been investigated—dPCR for measuring residual disease and NGS for detecting BCR‐ABL1 mutations [115, 116]. The emergence of more sensitive and accurate laboratory methods could, in the future, improve patient selection for TFR, possibly preceded by TKI dose reduction, in order to improve TFR success and minimize the TKI side effects.

## 9 Conclusion

Achieving sustained TFR remains a key goal in CML, requiring deeper insight into LSC biology, resistance mechanisms, and patient‐specific predictors. Future research should prioritize strategies to eradicate LSCs, develop reliable biomarkers for TFR success, and design combination therapies to increase the proportion of patients who can discontinue treatment safely.

TFR represents a shift toward precision and patient‐centered care, aiming to improve quality of life by eliminating the need for lifelong therapy. However, realizing this goal demands consideration of biological, clinical, and socioeconomic factors. By addressing the fundamental “W” questions (who, when, why, what, and where), this review provides a framework to advance TFR in CML. Continued progress will depend on refining predictive models, expanding access to novel therapies, and addressing global disparities to ensure equitable benefits for all patients.

## Author Contributions

All authors contributed to the manuscript and were involved in revisions and proofreading. All authors approved the submitted version.

## Conflicts of Interest

The authors declare no conflicts of interest.

## Data Availability

Data sharing is not applicable to this article as no new data were created or analyzed in this study.
